# IoT Big-Data Centred Knowledge Granule Analytic and Cluster Framework for BI Applications: A Case Base Analysis

**DOI:** 10.1371/journal.pone.0141980

**Published:** 2015-11-24

**Authors:** Hsien-Tsung Chang, Nilamadhab Mishra, Chung-Chih Lin

**Affiliations:** Department of Computer Science and Information Engineering, Chang Gung University, Taoyuan, Taiwan, ROC; Southwest University, CHINA

## Abstract

The current rapid growth of Internet of Things (IoT) in various commercial and non-commercial sectors has led to the deposition of large-scale IoT data, of which the time-critical analytic and clustering of knowledge granules represent highly thought-provoking application possibilities. The objective of the present work is to inspect the structural analysis and clustering of complex knowledge granules in an IoT big-data environment. In this work, we propose a knowledge granule analytic and clustering (KGAC) framework that explores and assembles knowledge granules from IoT big-data arrays for a business intelligence (BI) application. Our work implements neuro-fuzzy analytic architecture rather than a standard fuzzified approach to discover the complex knowledge granules. Furthermore, we implement an enhanced knowledge granule clustering (e-KGC) mechanism that is more elastic than previous techniques when assembling the tactical and explicit complex knowledge granules from IoT big-data arrays. The analysis and discussion presented here show that the proposed framework and mechanism can be implemented to extract knowledge granules from an IoT big-data array in such a way as to present knowledge of strategic value to executives and enable knowledge users to perform further BI actions.

## Introduction

An Internet of Things (IoT) big-data array can be defined as the large-scale organisation of IoT data into certain structural patterns in such a way as to ensure ease of use, ease of application, and ease of comprehension. The comprehension of IoT big-data arrays poses a challenge to current researchers due to the growing dimensions of IoT data-intensive applications for knowledge discovery [1]. The effective use and application of IoT big-data arrays requires the analysis of potential and explicit knowledge granules that can be successfully applied in numerous BI applications. Given the rapid growth of IoT applications in both commercial and non-commercial sectors, large-scale structured, semi-structured, and unstructured data arrays are produced daily. For example, the IoT evolutionary networks of Wal-Mart Stores, Inc., globally harvest approximately 2.5 petabytes of data per hour to store more than one million customer trade transactions [2]. Large IoT data arrays of this type are highly unstructured, ambiguous, and inadequate for the identification of tactical knowledge; nevertheless, some explicit knowledge granules are hidden within them. Thus, these issues necessitate further research to identify the tactical and explicit knowledge granules for effective corporate operations, such as planning, decision making, strategy building, and other operations, to develop intelligence for the BI applications.

In BI applications, the time to insight is slow, and the cost of insight is high. For these reasons, knowledge engineers are currently seeking new technologies and frameworks that can be used to cluster the potential knowledge granules and provide a basis for further cognitive decision making and organisational planning.

Machine learning techniques have been rapidly expanded to many areas of knowledge analysis and semantic knowledge analysis to support intelligence applications. Because the neuro-fuzzy system addresses more uncertainties than the standard fuzzified system in building an effective decision supportive knowledge framework for the above-listed applications, the implementation of neuro-fuzzy analytic architecture has been suggested for the analysis of knowledge granules [3–4]. The aim of applying neuro-fuzzy analytic architecture to implement the large-scale fuzzy-rule-based system is to produce high potential knowledge granules from IoT big-data arrays. Although some progress has been made in techniques for discovering the knowledge granules in IoT network platforms, there is still a lack of robust and effective techniques that can be used to discover the hidden potential knowledge granules present in large-scale IoT big-data arrays.

**Fig 1:**
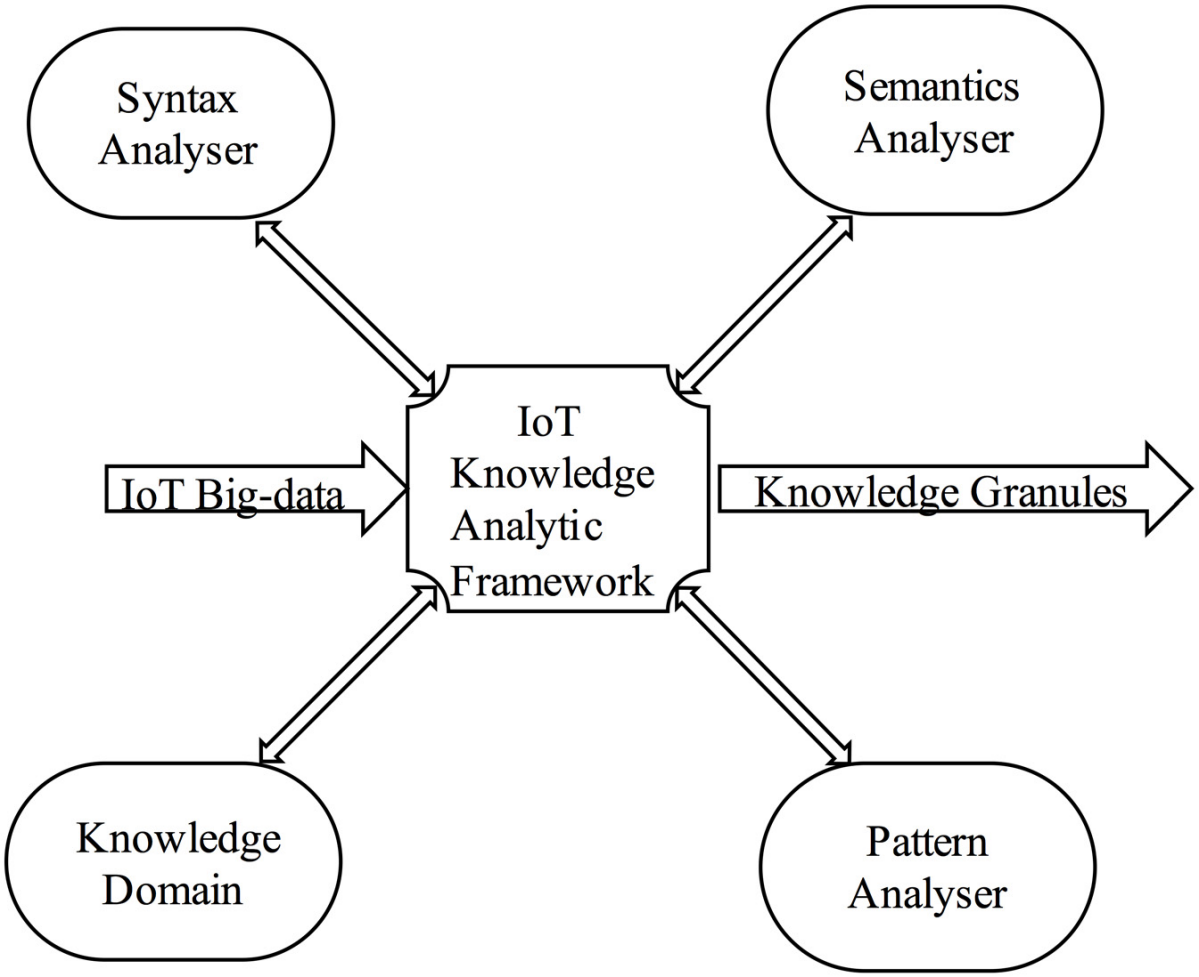
Alliances of an IoT knowledge analytic framework.

Four important components must be recognised when using neuro-fuzzy analytic architecture to implement a multi-rule-based system to discover hidden potential knowledge granules, as specified in Fig 1. Those components are the knowledge domain, pattern analyser, syntax analyser, and semantics analyser. While transforming the data/facts into knowledge granules, the syntax and semantics analysers must ensure that the facts are syntactically and semantically correct to produce knowledge granules of strategic value to present to executives and knowledge users to perform further cognitive actions.

The knowledge domain is an integrated, knowledge-based system or an expert system that uses the multi-rule based system to generate knowledge granules. As the IoT big-data array faces the challenge of high ambiguity, a pattern analyser may make it possible to increase the effectiveness of the multi-rule based system, thereby generating high performance, error tolerance, and reliable knowledge granules. The syntax and semantic analysers play a critical role in permitting very large-scale syntax and semantic event processing over a multi-rule-based system.

In our work [5], we discuss an in-network knowledge collection framework for a BI application, i.e., a safety-critical industrial application. In this work, we suggest an abstract of a data-to-knowledge (D2K) migration framework that uses a neural network (NN) architecture to produce knowledge of tactical value from IoT data to regulate the industrial application. However, in actual implementation of a BI-industrial application, the training and testing of a monotonic NN-filter is associated with high computational demands, and some time frames may not be adequate for promoting knowledge collection even after successful training and testing is completed.

In this work, we further modify the D2K migration architecture through a fuzzy inference system that has more industrial applicability than the previous approach. We study four unified approaches that can be widely implemented for time-critical industrial applications; these approaches are knowledge discovery strategies implemented via a neural network (KDS-NN), a genetic algorithm (KDS-GA), standalone data-mining algorithms (KDS-DM), or a fuzzy inference system (KDS-FIS). We empirically estimate the performance dimension matrix of the three main approaches—KDS-NN, KDS-GA, and KDS-FIS. We perform a relative performance evaluation based on approximate defuzzification reasoning and observe that the KDS-FIS approach is superior to other approaches in terms of its reasoning mechanism, knowledge analytic efficiency, decision-making capability, computational efficiency, and industrial applicability, especially for a safety-critical industrial application.

In the present work, we consider only the structured data that are to be handled in our proposed architectures. However, in many potential IoT-regulated BI applications, data volume is typically large-scale and tends toward big-data applications in which IoT big data are an integration of structured, semi-structured, and unstructured data with high complexity, redundancy, and ambiguity [6]. Therefore, to discover the potential knowledge granules from an IoT big-data array for a BI application, we suggest implementing a neuro-fuzzy analytic architecture rather than a standard fuzzified approach to discover and structure the complex knowledge granules. Furthermore, we implement an enhanced knowledge granule clustering (e-KGC) mechanism that is more elastic than previous techniques in clustering the tactical and explicit complex knowledge granules from large-scale IoT big-data arrays.

The remainder of this paper is organised as follows. In Section **2**, a discussion of related studies is presented. The knowledge granules analytic and cluster framework from IoT big-data arrays is primarily highlighted in section **3**. Section **4** focuses on the analysis and discussion of the proposed framework; and finally, section **5** marks the conclusions of this paper and future work.

## Associated Studies

In this section, we discuss allied studies related to the analysis and clustering of knowledge granules from IoT big-data arrays associated with some BI applications. Here, we consider IoT big data to be large-scale sensor data, RFID data, and the data of wearable and non-wearable sensing devices. The IoT big-data array may be capable of handling the increasing volume of large-scale data produced by numerous IoT applications. The IoT big-data problem raises several challenges: capture, storage, search, analysis, and visualisation [7]. The analysis of knowledge granules is based on the degree of analysis and on the explorations of IoT big data, especially for IoT-based BI applications; the intervention of such analysis and exploration leads to the development of BI that can potentially be used to measure and analyse the various business resources [8–9]. The BI application ensures the analysis and measurement of the consumer’s thoughts, behaviours, relationships, buying attitudes, choices, and many more parameters that form the backbone of effective strategy building, business operations management, customer relationship management, and other business operations [10]. Normally, IoT big data are highly unstructured, ambiguous, inconsistent, and incomplete, thereby creating many hazards in the analytic of potential knowledge granules related to BI applications. This creates potential challenges for data mining and knowledge engineering researchers attempting to identify the potential knowledge granules that can be effectively clustered for further analysis and exploration [11–12]. The real-time structuring of knowledge granules from IoT big data are typically problematic due to their strict time constraints, large data volume, data heterogeneity, and asynchronous incoming data streams. Thus, the work in [13] emphasises framing the knowledge granules obtained from IoT big data in terms of logical formulas, graphs, strings, and other structures. The multi-rule based system suggests new structures and patterns that can be used to improve the real-time structuring of knowledge granules for various BI applications. Several works implement a fuzzy associated rule-based system for numerous BI applications, such as monitoring falls in the elderly, traffic congestion, gas metal arc welding, and some bio-processing applications [14–17]. The type-2 neuro-fuzzy associated multi-rule based system has a greater degree of precision and tolerance in terms of big-data analysis, knowledge granule analytic, and visualisation than a standard fuzzified approach. Thus, the work in [18–21] implements the type-2 neuro-fuzzy approach for modelling and regulating various BI applications. In a multi-rule based system, the syntax and semantics associated analysis is a major factor in identifying high-performance, error-tolerant, and reliable knowledge granules [22].

Prior to the design of our proposed framework, we discuss some works associated with multi-criteria decision-making systems, frameworks, and tools and others that have previously been applied to different BI services and can be functional in the IoT big data environment. In the advanced IoT big data environment, multi-criteria decision-making processes accompanied with progressive BI-service applications use several diversified knowledge analytic technologies, such as neuro-fuzzy-GA embedding technology, deep learning and emerging deep learning technologies, and extreme learning machine (ELM) technology, to boost the business security strategies, and almost all service applications require the structural and semantic knowledge analytics from large-scale IoT data that are distributed across the business environment. In [23], fuzzy TOPSIS (a multi-criteria decision-making technique for order preference by similarity) was used to implement a fuzzy mechanism to control the business resources and IoT data/information flow for the BI-service application. The integrated fuzzy multi-objective programming (MOP)-based multi-criteria decision-making process for BI-service application is discussed in [24]. Other studies focus on standalone fuzzy/GA/neural-based knowledge analytic mechanisms to boost the multi-criteria decision-making systems for BI-service applications [25–27]. Furthermore, we also study BI-service applications that implement either fuzzy-based or fuzzy-GA or neuro-fuzzy-based knowledge analytic technologies to boost the multi-criteria decision-making process used to construct a business security strategy of a business-to-consumer (B2C) organisation selling products and services [28].

Based on the foregoing studies and analyses, we were motivated to design a KGAC framework for the effective analytic and clustering of knowledge granules from IoT big-data arrays associated with BI applications. In our KGAC framework, we also implement a type-2 neuro-fuzzy associated multi-rule based system to improve the identification of potential implications of knowledge granules in BI applications. The ontology-based knowledge clustering approach has captured interest in IoT big data in regard to accumulating the large-scale depository of semantic knowledge to be applied to regulating and actuating BI applications [29–30]. We also implant an e-KGC mechanism in the KGAC framework for the effective clustering and sub-clustering of knowledge granules based on associated characteristics and fitness value.

## Knowledge Granule Analytic and Cluster (KGAC) Framework

**Fig 2:**
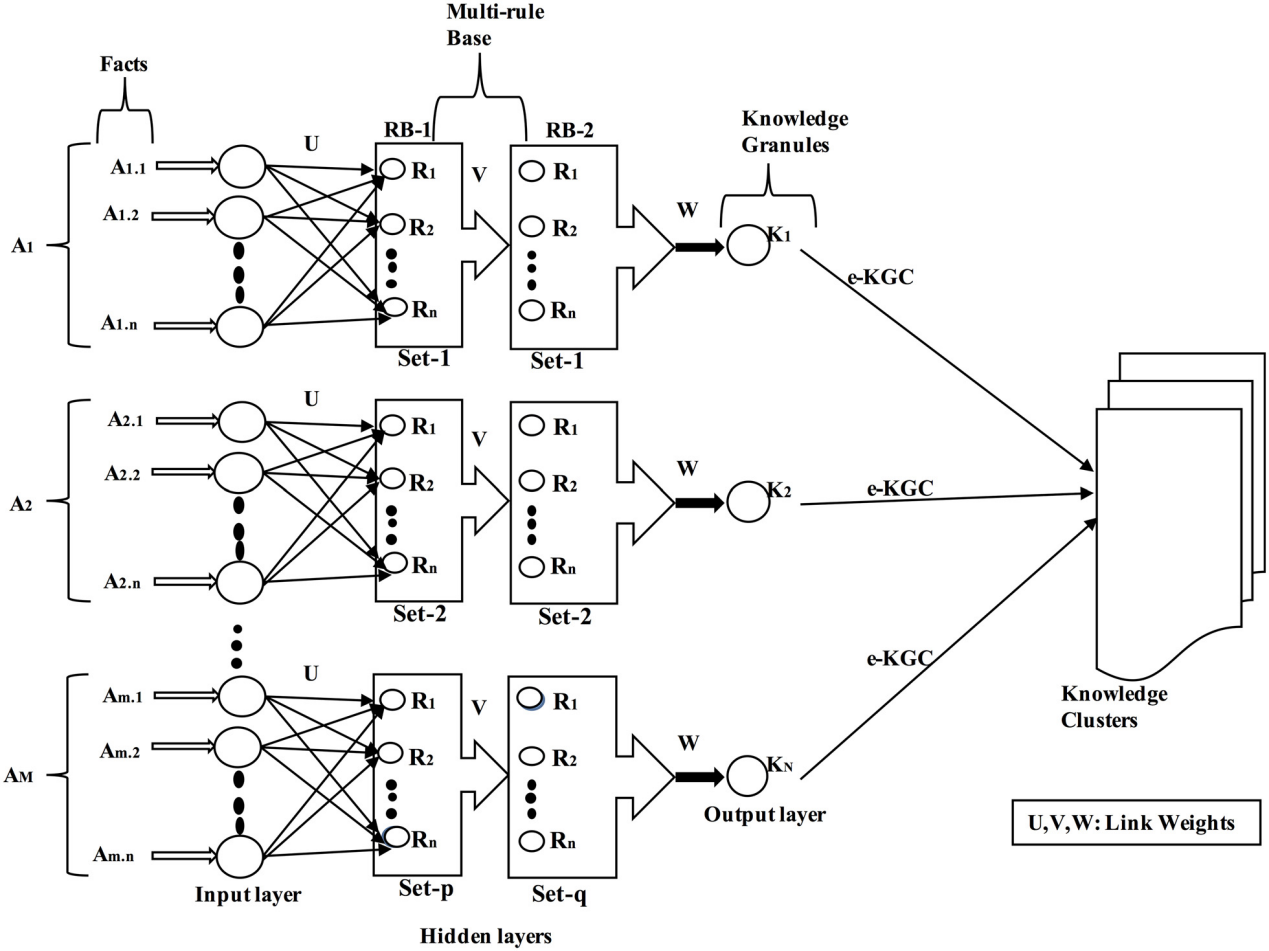
Neuro-fuzzy representation of a KGAC framework for a BI application.

In this section, we discuss the KGAC framework in detail. The facts are organised in the form of a big-data array within the multi-rule based system to explore the knowledge granules. The knowledge granules are then mapped to the knowledge clusters (KCs) for further analysis and exploration. The neuro-fuzzy analytic architecture is key to this analytic; furthermore, the clustering of knowledge granules can be performed through an e-KGC mechanism. Fig 2 describes a neural representation of knowledge granule analysis and a cluster framework for an IoT big-data array in which the multiple rule bases are enclosed within the hidden layers of a self-adoptive neural network. In our framework, we consider two rule bases—RB-1 and RB-2. RB-1 consists of p sets of rules; however, RB-2 consists of q sets of rules such that each set consists of n number of sub-rules that specify R_1_, R_2_,…,R_n_. In the input layer, we consider a large-scale IoT big-data array in which the data/facts are organised with a dimension of ‘m.n’. In the output layer, at a particular time, N knowledge granules are produced and stored in the KCs. Let U, V, and W be fuzzy weights between the layers such that the corresponding weight matrix can be estimated through the fitness functions y = f (F, R, Ɵ), where y is the output of the weight matrix, F is the input fact array, R is the rule array, and Ɵ is the corresponding fitness value. The fitness selection is performed by choosing a value for Ɵ that best fits the rule array with the corresponding fact array.

Let, for instance, y_1_ = f (F_1_, R_1_, 7) on a fitness scale of 1–10; this shows that the fact array F_1_ is 70% fit with rule array R_1_ to perform the analytic of knowledge granules, i.e., the knowledge analytic precision (KAP) is 0.7. In Fig 2, the configuration of the KGAC framework is considered to be “mn: n^p^: n^q^: N”, where mn is the total number of input neurons, n^p^ is the total number of neurons present in the first hidden layers, n^q^ is the total number of neurons present in the second hidden layer, and N is the total number of output neurons. As described in Table 1, the input layer of the KGAC framework is responsible for fetching the IoT big-data array to the multi-rule base and for performing syntax and semantics analysis operations to ensure that the data/facts are sufficiently syntactically and semantically correct to be used in the knowledge granule analytic process.

**Table 1:** Responsibilities of the individual layers in a KGAC framework.

Layers	Responsibility
Input layer	Syntax and semantics analysis
1st hidden layer	Pattern analysis
2nd hidden layer	Expert system support
Output layer	Knowledge granule accumulation
Cluster layer	Clustering of knowledge granules

The first hidden layer of the KGAC framework acts as a pattern analyser to identify and suggest the correct patterns in the big-data array; however, its structure and configuration differ from application to application. The problem consists of designing an expert system for the second hidden layer of the KGAC framework that consists of predefined sets of rules that can be used to impart cognitive judgements and decision-making for achieving desired intelligence.

A knowledge domain inherits the features of a natural language processing model, conceptual dependency model, and logical inference model and predicates a model that can be used to construct a knowledge-based information system, expert system, or rule-based system. The expert system consists of rule-based, mathematically based, statistically based, textually based, case reasoning-based, and structure-based contexts to incorporate human historical experience, analytical skills, logical reasoning, and innovative ideas to ensure an effective knowledge granule analytic system. The output layer of the KGAC framework mainly accumulates the knowledge granules and sends them to the cluster layer for proper associations through an e-KGC mechanism in such a way that homogeneous knowledge granules must be in their respective clusters. Here, we elaborate the formulation of a multi-rule-based system and an e-KGC mechanism of the KGAC framework for prospective implementations.

### 3.1. Formulation of a multi-rule based system

**Fig 3:**
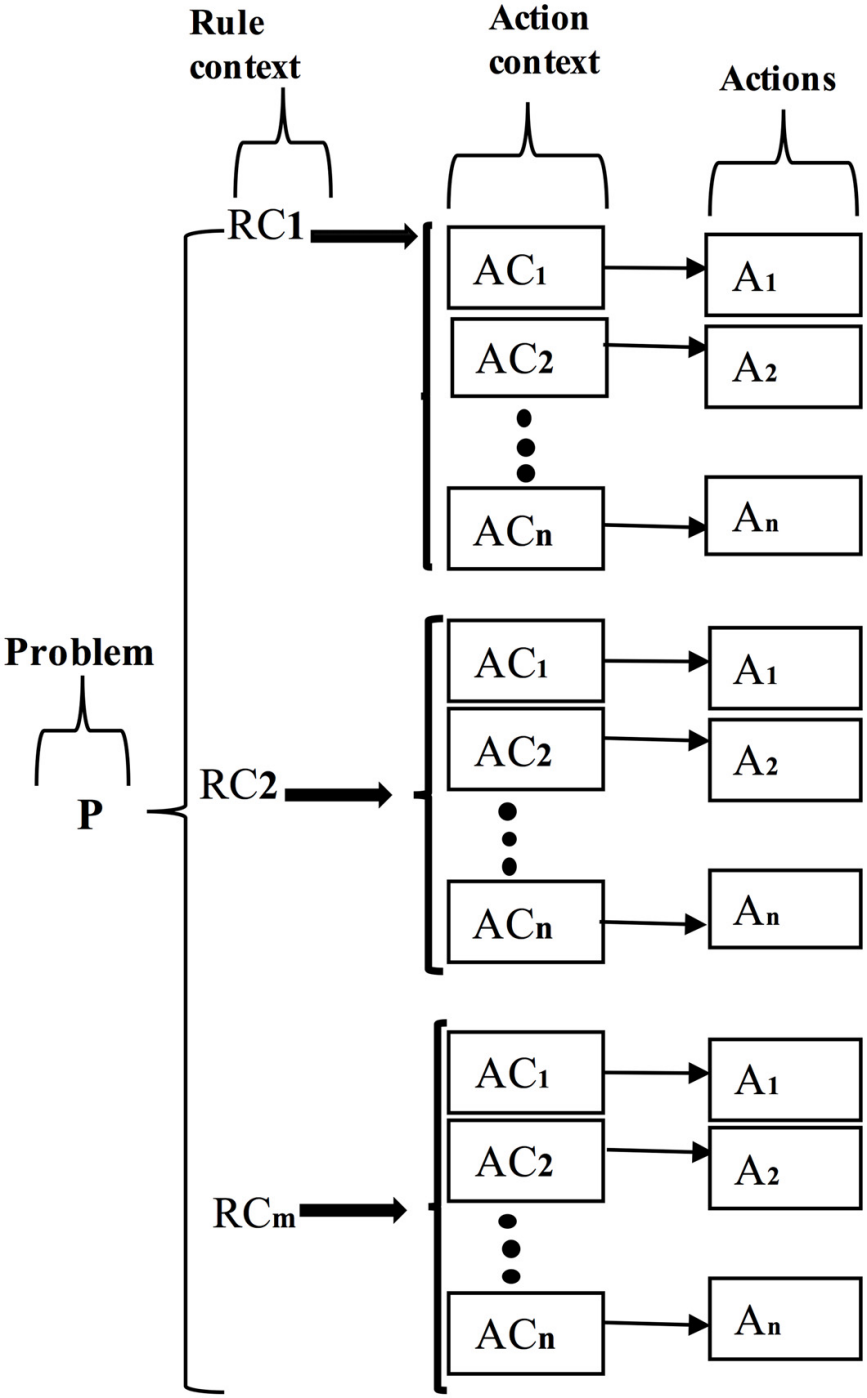
Structure of a multi-rule base.

The multi-rule based system can be used to represent highly multifaceted decisions for numerous BI applications [31–34]. Human decision making is purely internal and is not sufficiently simple to be represented as a set of rules to determine the internal control flow. However, some knowledge representation techniques can be successfully used in different applications in which the knowledge granules are represented as a set of rules. The structure of a typical multi-rule base is graphically represented in Fig 3. Normally, four queries are to be effectively synchronised while designing any multi-rule-based system:

1. The precise definition of the BI problem.
2. The context in which the BI rule is implemented.
3. The context from which the specified BI action/conclusion was derived.
4. The feasibility of applying the specified BI action/conclusion.

Normally, the decision of any BI application follows a sequence of production rules in which each production rule can be represented in an IF-THEN arrangement. Several problems are associated with a BI application; let P be an allied problem. The problem P consists of several rule contexts; furthermore, each rule context comprises several action contexts in such a way that each individual action context is linked with finite action. The rule and action context may be defined as the background, the situation, the environment, and the circumstances under which the rule and action can be effectively implemented toward regulating the application. A sample set of production rules is presented in Table 2. The multi-rule based system over BI problem P can generate a maximum of mXn rules and actions for m numbers of the rules context and n numbers of the actions context.

**Table 2:** Sample set of production rules for a KGAC framework.

Sequence	Rules
RC_1_.R_1_	IF P AND RC_1_ AND AC_1_ THEN A_1_
RC_1_.R_2_	IF P AND RC_1_ AND AC_2_ THEN A_2_
RC_1_.R_n_	IF P AND RC_1_ AND AC_n_ THEN A_n_
RC_2_.R_1_	IF P AND RC_2_ AND AC_1_ THEN A_1_
RC_2_.R_2_	IF P AND RC_2_ AND AC_2_ THEN A_2_
RC_2_.R_n_	IF P AND RC_2_ AND AC_n_ THEN A_n_
RC_m_.R_1_	IF P AND RC_m_ AND AC_1_ THEN A_1_
RC_m_.R_2_	IF P AND RC_m_ AND AC_2_ THEN A_2_
RC_m_.R_n_	IF P AND RC_m_ AND AC_n_ THEN A_n_

### 3.2. e-KGC mechanism

Definition: A mechanism used to regulate the resemblance of knowledge granules to be found in identical clusters and sub-clusters based on the characteristics and fitness values associated with it.

The definition of an e-KGC mechanism specifies that each knowledge granule must be associated with a fitness tag, where the estimated fitness value *θ* is present. Based on the characteristics of the knowledge granule, the initial mapping is performed for a cluster, and based on the fitness value *θ*; the next level mapping is performed for the respective sub-clusters under that cluster.

**Fig 4:**
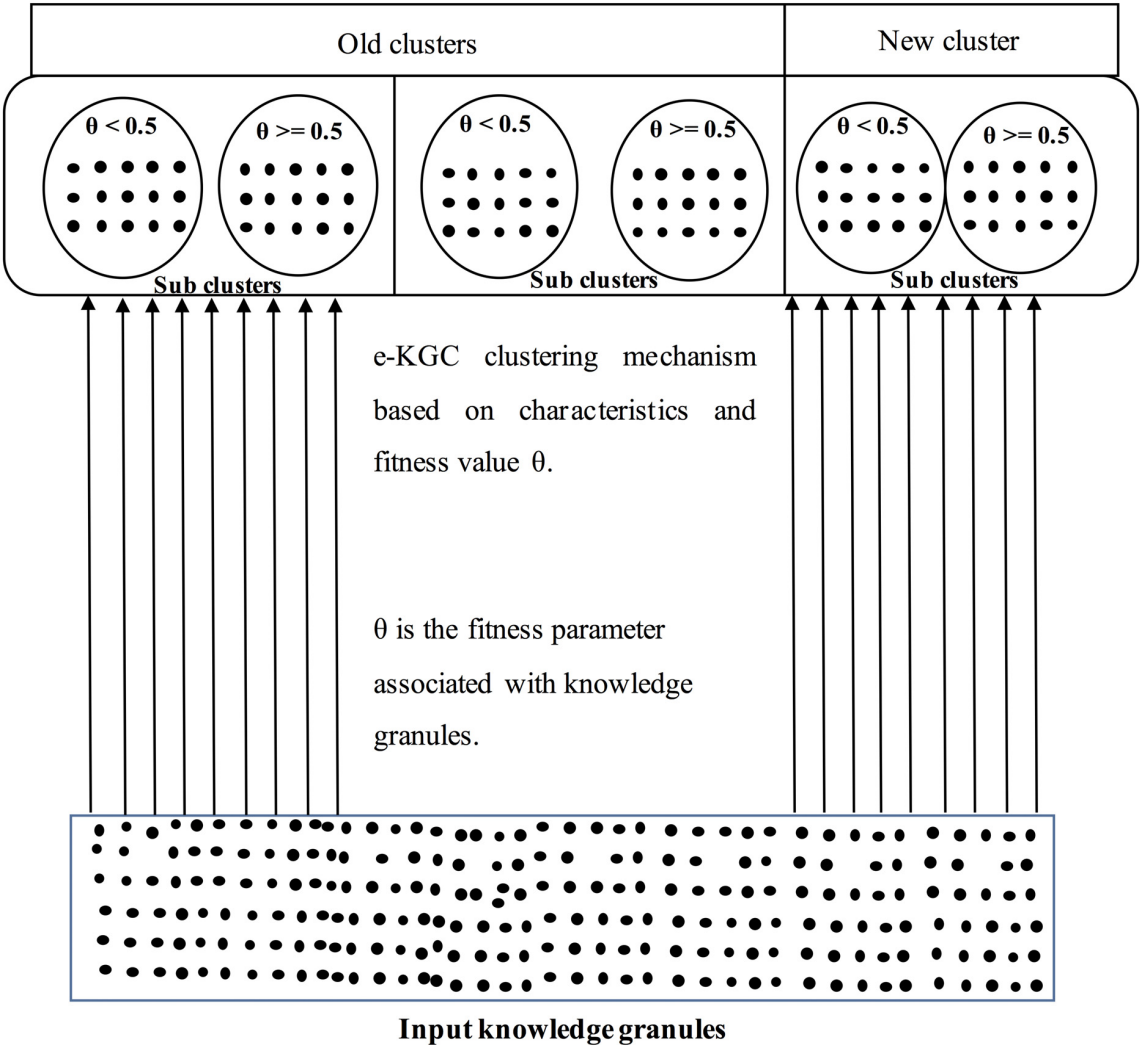
Architecture of the e-KGC mechanism.

Several text-based or feature-based clustering algorithms have been proposed by data mining researchers [35–37]. However, we present a simple knowledge-clustering mechanism that is based on the associated θ values. Fig 4 describes the architecture of an e-KGC mechanism for granular-level knowledge clustering based on the fitness value. Two clusters are said to be identical if they possess knowledge granules with homogeneous characteristics. The knowledge granules are mapped to the individual cluster based on the characteristics. Within a cluster, the sub-clusters are maintained in such a way that the fitness of the knowledge granules can be easily identified. Within a cluster, let K_1_ be a knowledge granule such that K_1_ is mapped into sub-cluster (θ < 0.5) if and only if θ (K1) < 0.5; else, K_1_ is mapped into sub-cluster (θ > = 0.5). The θ values of clusters and sub clusters are dynamically estimated through quantifying the outliers that are present inside, and more outliers present inside the clusters and sub-clusters degrade θ values. We discuss the implementation details in the analysis and discussion section through a case analysis to ensure further exploration.

### 3.3. Analytical implementation

The implementation of neuro-fuzzy implementation helps improve the KGAC framework so that it can identify and cluster knowledge granules with the maximum fitness values; this clusters the best-fit knowledge granules for the configuration of a multi-rule based system to regulate BI applications. As specified in the KGAC framework, the IoT big-data array can be configured in a two-dimensional matrix (A_k_) having dimensions mXn (k ϵ mXn), and the matrix is represented as a fact matrix that can be used to scale and organise the IoT big data.

Therefore, A_k_ =

$$\left|\begin{array}{cccc} A_{11} & A_{12} & A_{1j} & A_{1n}\\ A_{21} & A_{22} & A_{2j} & A_{2n}\\ A_{31} & A_{32} & A_{3j} & A_{3n}\\ \begin{array}{l} A_{i1}\\ A_{m1} \end{array} & \begin{array}{l} A_{i2}\\ A_{m2} \end{array} & \begin{array}{l} A_{ij}\\ A_{mj} \end{array} & \begin{array}{l} A_{in}\\ A_{mn} \end{array} \end{array}\right|$$

Now, by considering the fitness value θ and array elements, the fuzzy equations can be derived as follows:

$${\hat{A}}_{k}=\frac{\sum \mu_{\hat{A}}\left(k,\theta \right)}{\theta };where\;\theta =\left[0,1\right]$$ (1)

A^1= A11θ1+A12θ2+A13θ3+A1nθn (2)

A^1= A21θ1+A22θ2+A23θ3+A2nθn (3)

A^1= Am1θ1+Am2θ2+Am3θ3+Amnθn (4)

Now, based on Eqs (2), (3), and (4), the fact matrix of the KGAC framework can be expressed as follows i.e., (A_k_ / *θ*_*n*_ *)*. Hence, Ã_k_ =

|A11θ1A12θ2A1jθjA1nθnA21θ1A22θ2A2jθjA2nθ2A31θ1A32θ2A3jθjA3nθnAi1θ1Am1θ1Ai2θ2Am2θ2AijθjAmjθjAinθnAmnθn|

Now, the problem consists of computing fitness value θ for Ã_k_ through the neural representation of the KGAC framework. We represent the KGAC framework as a partially connected feed-foreword multi-layer neural network, which is self-organising in its configuration to act as an unsupervised multi-rule-based agent. The abbreviations used for the KGAC framework to explain the internal functions and operations are described in Box 1.

**Box 1. Abbreviations of the KGAC framework**

I_i_ = Input of input layer, O_i_ = Output of input layer,

I_h1_ = Input of hidden layer-1, O_h1_ = Output of hidden layer-1,

I_h2_ = Input of hidden layer-2, O_h2_ = Output of hidden layer-2,

I_o_ = Input of output layer, O_o_ = Output of output layer.

I_i_ accepts the IoT big-data array as a normalised two-dimensional fact matrix, performs the syntax and semantics analysis, and produces the enriched fact matrix through O_i_.

I_h1_ accepts the fact matrix and performs the pattern analysis operations to identify and resolve the ambiguous, inconsistent, and incomplete patterns according to the norms and standards of the structural patterns that have been predefined for the BI applications. O_h1_ ensures production of high-quality structural patterns that can effectively participate in the analytic of knowledge granules. The pattern computations may be performed using the following standard equations.

$$T=\sum_{i=1}^{n}\left({Ã}_{k1}\left(i\right)\right).{Ã}_{k}\left(i\right)$$ (5)

where T is the connectionist matrix, Ã_k1_ is the transpose of Ã_k_, and n is the number of input patterns.

$$Generated\;Pattern\left({\hat{A}}_{j}"\right)=f\left({\hat{A}}_{j}\cdot T_{ij},\;{\hat{A}}_{j}\right)=f\left(\alpha ,\beta \right)$$ (6)

where j is the dimensions of input patterns. The computation of f (α, β) depends on the value of α, i.e., f (α, β) = 1 if α > 0, f (α, β) = β if α = 0, and f (α, β) = -1 if α < 0.

I_h2_ accepts the approximated patterns and interacts with the knowledge domain to empirically determine the fuzzified fitness value *θ*. The degree of truthfulness and accuracy can also be measured in this level to empirically estimate the approximate value of *θ*. In this manner, O_h2_ produces the knowledge granule patterns associated with θ to distinguish the best-fit knowledge granules from the IoT big-data array to present knowledge of strategic value to executives and knowledge users, enabling them to perform further cognitive actions.

I_o_ accepts those knowledge granules and then passes them to O_o_ for the purpose of accumulation. This layer periodically sends those knowledge granules to the clustering layer for storage, analysis, and further exploration. As discussed earlier, the cluster layer mainly uses the e-KGC mechanism to store those knowledge granules. The output layer also has a critical role in synchronising with the cluster layer with the aim of minimising the critical loss of knowledge granules for BI applications. In the cluster layer, the semantic associations and linkage of knowledge granules can be visualised as a basis for further cognitive action.

**Fig 5:**
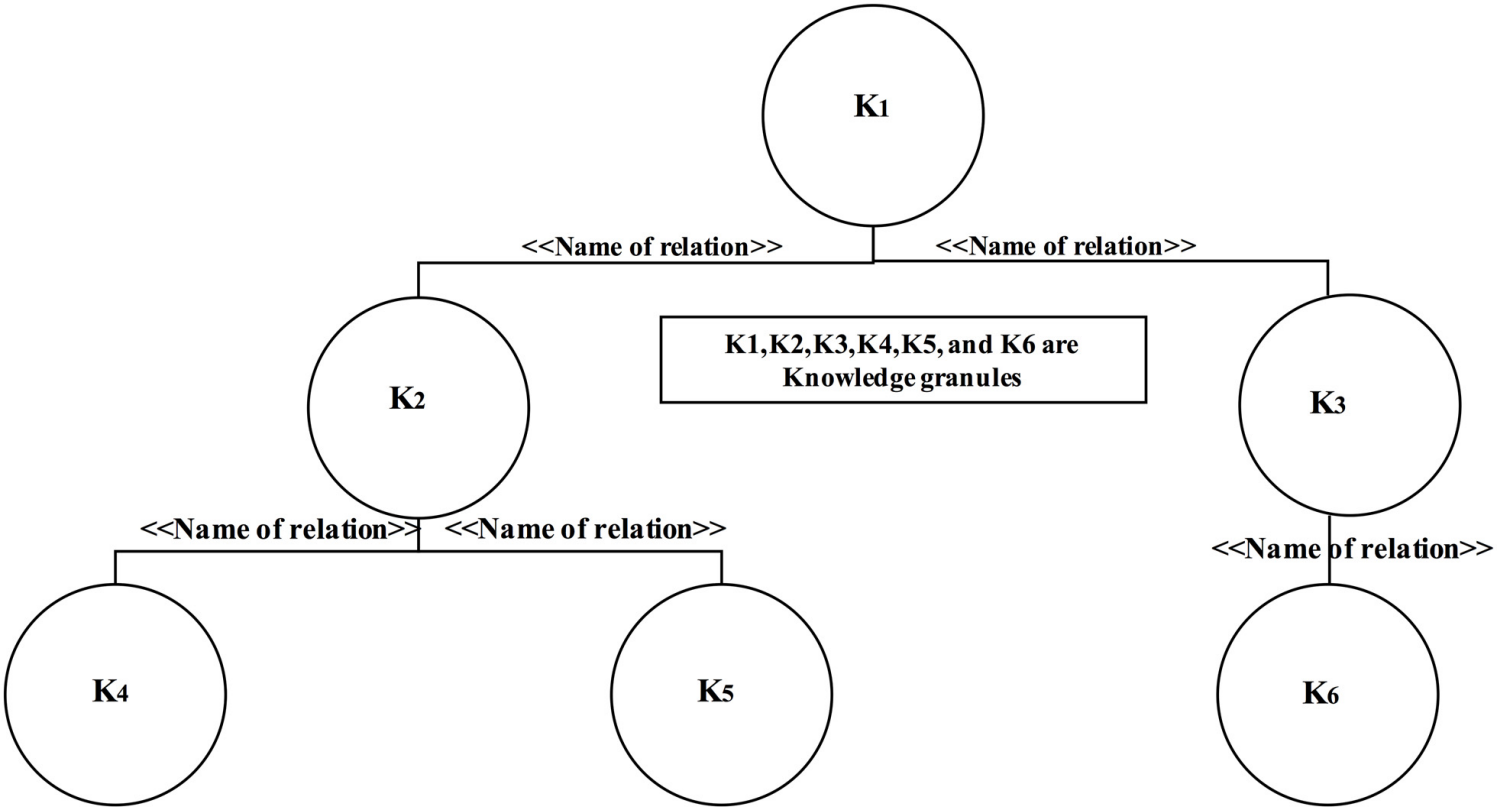
Semantic associations of knowledge granules within a KC.

Here, we give an example of the semantic association of knowledge granules for better clarification; a sample is presented in Fig 5. Let us assume that K_1_, K_2_, K_3_, K_4_, K_5_, and K_6_ are six knowledge granules that are semantically associated in a hierarchical structure according to well-defined semantic relationships. Within the structure, K_4_, K_5_, and K_6_ are mutually independent and have no relationships between them; the situation is the same for K_1_ and K_3_. However, K_1_ is semantically associated with K_2_ and K_3_ and is transitively associated with K_4_, K_5_, and K_6_. From the IoT big-data array, a large number of such semantic associations of knowledge granules can be scaled into a certain structural pattern to ensure ease of use, ease of application, and ease of comprehension. This type of semantic analysis of the knowledge granules helps executives and knowledge users develop intelligence for BI applications.

Prior to performing large-scale structural analysis, we visualise (Fig 5) a sample schema of semantic associations of knowledge granules within a KC in a hierarchical manner. Such semantic associations may form the backbone of the large-scale structural analysis of knowledge granules that have complex semantic relationships. Thus, in the context of analysis and discussion, we propose a larger-scale organisation of knowledge granules in the form of knowledge sub-clusters (KSCs) in which each sub-cluster consists of a hierarchical structure depicting the semantic associations of the desired knowledge granules for the specific application.

## Analysis and Discussion

This work analyses the implementation of our proposed KGAC framework through a neuro-fuzzy analytic architecture. Here, we may consider the fuzzy associated data sets for a BI application to analyse and visualise the potential knowledge granules that can participate in effective functional operations of the business [38–39]. We broadly classify the analysis and discussion part into two phases. Phase 1 includes the structural analysis of large-scale sub-cluster organisations to accommodate the IoT big-data array. Phase 2 highlights the computational analysis used to empirically study the performance of our KGAC framework applications.

### 4.1. Phase 1 (Large-scale Structural Analysis)

**Fig 6:**
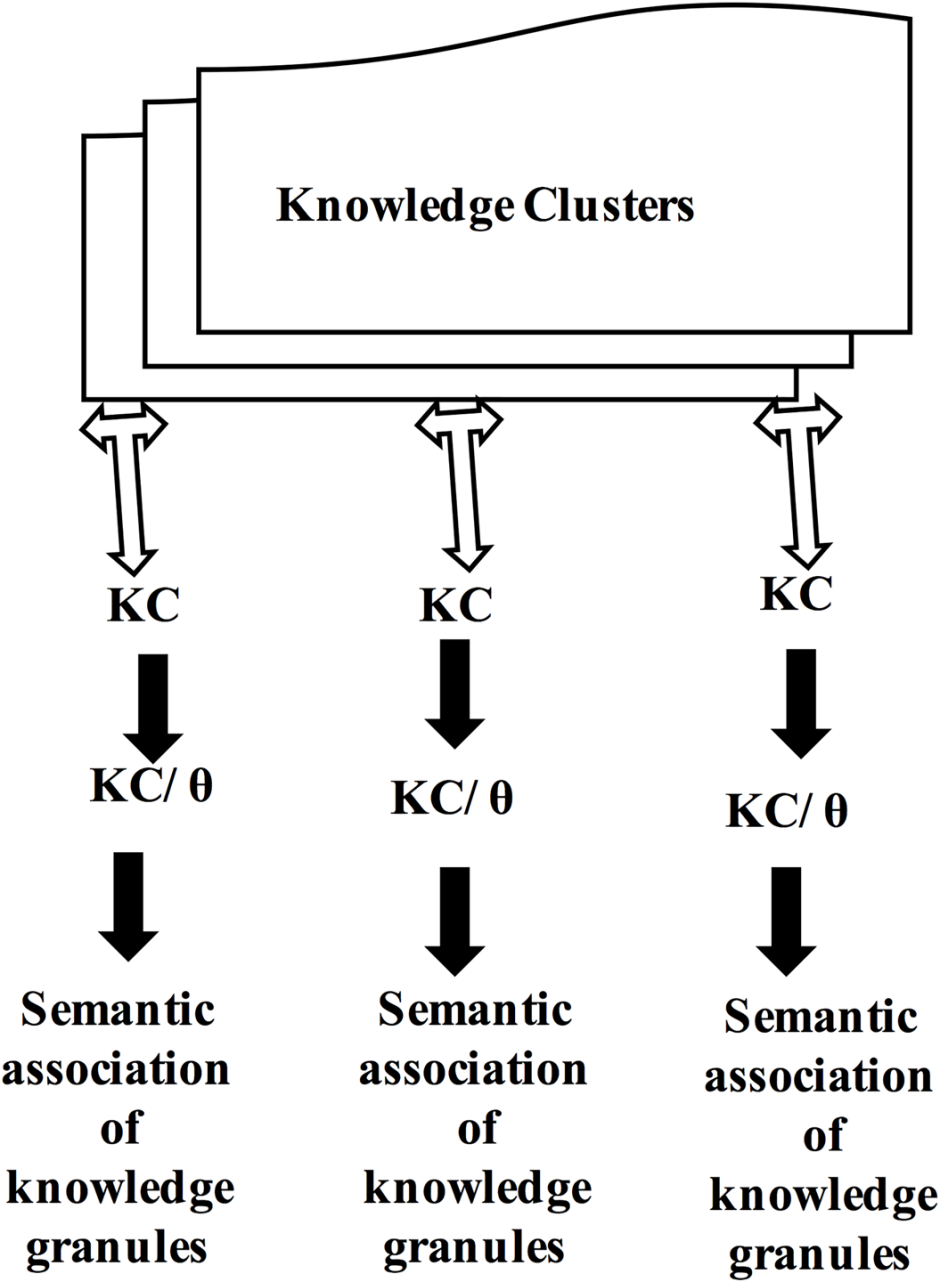
Basic correlation of KCs.

In this phase, we discuss the structural exploration of large-scale clusters and sub-cluster organisation along with the semantic linkage analysis used to cope with the IoT big-data array. The basic correlation of the knowledge set (KS), in which each KC is logically divided into a number of KCs such that each individual cluster is correlated with a KS, is described in Fig 6. Each KC consists of semantic associations of knowledge granules; the fitness vector θ is associated with an individual KC so that the specific KS will participate in driving the application based on critical requirements. The progressive computer architecture supports the architectural base of the multi-dimensional IoT big-data array for many parallel machines and can be used for large-scale computations and knowledge granule analysis in numerous BI applications.

**Fig 7:**
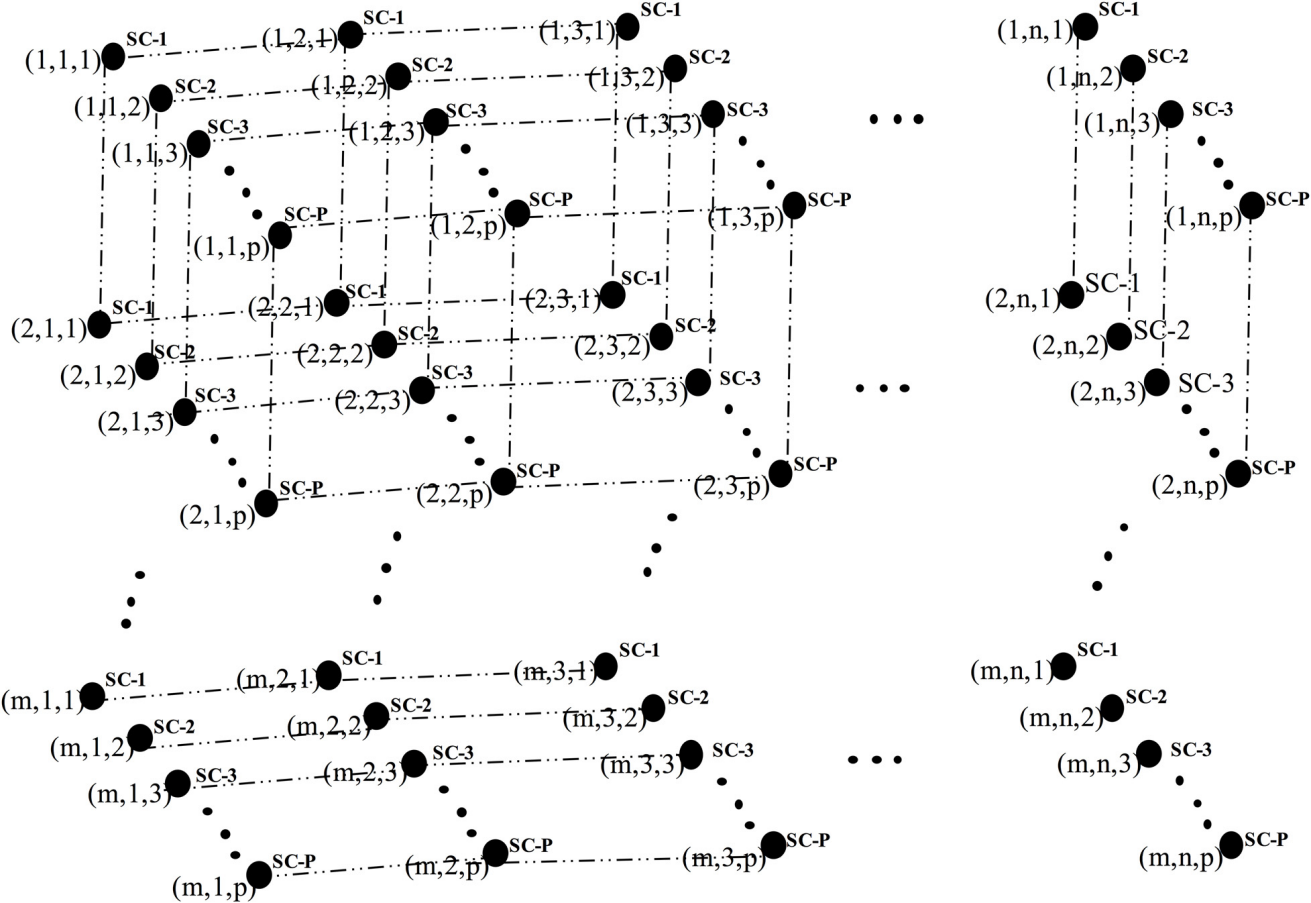
Large-scale organisation of KSCs in a k-dimensional p-sided IoT big-data array, where k = 3 and p is the number of sub-clusters associated with each KC.

**Fig 8:**
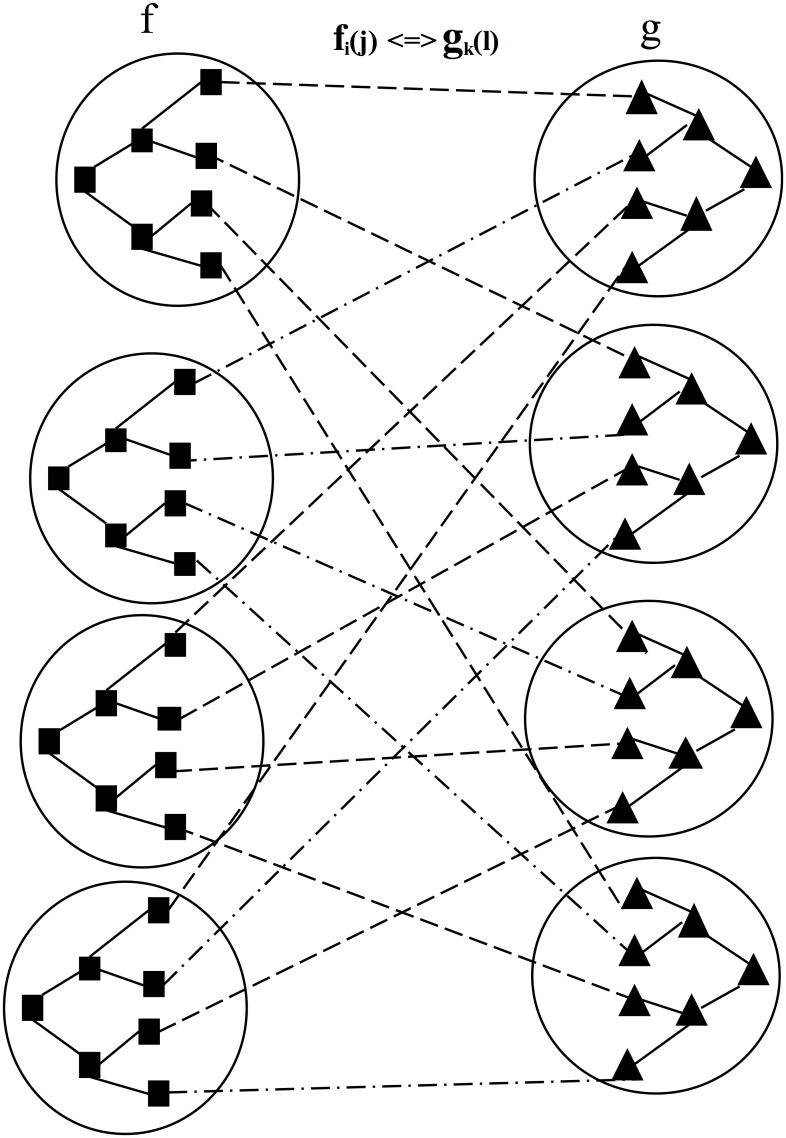
Large-scale semantic linkage analysis of knowledge granules in between the KSCs.

In Fig 7, we describe the large-scale organisation of KSCs in a k-dimensional p-sided IoT big-data array, where k = 3 and p is the number of sub-clusters associated with each cluster. Some analysis has been conducted here to compute the diameter and total KS. Here, diameter = k (p-1) and total KS = p^k^. The higher-dimensional array of KCs and KSCs organises the knowledge granules in accordance with standard principles and strategies that vary across numerous BI applications. In Fig 8, we describe a large-scale semantic linkage analysis of knowledge granules in two KCs. Fig 5 defines the interrelationships of knowledge granules within a sub-cluster. A large-scale extension of Fig 5 is presented in Fig 8 for the purpose of focusing on both the intra and interrelationships among the sub-clusters.

Let f and g be two clusters of knowledge granules such that f_i_(j) < = > g_k_(l) defines the semantic linkage between the j_th_ knowledge granule of the i_th_ sub-cluster of cluster f and the lth knowledge granule of the kth sub-cluster of cluster g and < = > defines the semantic mapping linkage between them.

By analysing the large-scale associated semantic linkage, standard semantic associations between clusters f and g can be discovered. The equation is written as follows:

$$f_{i}\left(j\right)\Leftrightarrow g_{k}\left(l\right);for\;i,j,k,l=1,2\;\cdots n$$ (7)

Eq 7 can be analysed to obtain the maximum possible number of associations between clusters f and g. For a specific semantic knowledge granule association scenario, let the values i = j = k = l = 4; accordingly, the possible associations for the above scenario of Fig 8 are described as follows:

$$f_{1}\left(1\right)\Leftrightarrow g_{1}\left(1\right)$$ (7.1)

$$f_{1}\left(2\right)\Leftrightarrow g_{2}\left(1\right)$$ (7.2)

$$f_{1}\left(3\right)\Leftrightarrow g_{3}\left(1\right)$$ (7.3)

$$f_{1}\left(4\right)\Leftrightarrow g_{4}\left(1\right)$$ (7.4)

$$f_{2}\left(1\right)\Leftrightarrow g_{1}\left(2\right)$$ (7.5)

$$f_{1}\left(2\right)\Leftrightarrow g_{2}\left(2\right)$$ (7.6)

$$f_{2}\left(3\right)\Leftrightarrow g_{3}\left(2\right)$$ (7.7)

$$f_{2}\left(4\right)\Leftrightarrow g_{4}\left(2\right)$$ (7.8)

$$f_{3}\left(1\right)\Leftrightarrow g_{1}\left(3\right)$$ (7.9)

$$f_{3}\left(2\right)\Leftrightarrow g_{2}\left(3\right)$$ (7.10)

$$f_{3}\left(3\right)\Leftrightarrow g_{3}\left(3\right)$$ (7.11)

$$f_{3}\left(4\right)\Leftrightarrow g_{4}\left(3\right)$$ (7.12)

The evolution of computer architecture in a parallel programming platform may efficiently regulate the quick processing of higher- dimensional IoT big-data arrays and thereby contribute to the analytic of knowledge granules. For a p-sided array, the dimension k can also be computed as k = log p, but contemporary processors can efficiently handle the higher-dimensional array, i.e., it is limited to three.

### 4.2. Phase 2 (Computational Analysis)

In this phase, comprehensive computational analysis is used to empirically study the performance of our KGAC framework implementations. A number of computational intelligence approaches, such as the fuzzy approach, neural approach, type-1 neuro-fuzzy approach, and type-2 neuro-fuzzy approach, are available to implement the functional processes of KGAC framework; however, the type-2 neuro-fuzzy analytic approach is more effective in offering greater tolerance and better at addressing the uncertainties actually encountered in BI applications [40–41]. The functional and operational analysis of our KGAC framework is mapped to a neuro-fuzzy analytic architecture to empirically estimate the KAP. The KAP is directly proportional to the fitness value θ. Higher θ values associated with a data set lead to the greater KAP of the KS and a high degree of appropriateness accuracy with respect to BI applications.

#### 4.2.1 Case Analysis

In this case analysis, we talk over a business intelligence case that supports to construct a business security strategy of a B2C organisation selling products and services. Consider an example case of a B2C organisation, such as Flipkart.com or amazon.com that trusts on an e-commerce application to sell their products and services directly to the consumers. Such organisations rely heavily on the progressive consumer’s buying behaviour to improve their business security strategy. The organisation can use consumers’ shopping experience, views on existing service reliability, product quality, and trade policies rated on a scale of 0–10 in terms of consumers’ response that will be recorded and redirected to a structured feedback database for further knowledge analysis and business use. The B2C e-commerce organisations also take the advantage of advanced IoT-based distributed knowledge analytics to transform the consumer’s end analytics into goldmines of business worth. The IoT big data, through coordinated machine learning and integration of different data sources and actions, creates several challenges and opportunities for the progressive e-business scenarios. The consumers’ end analytic is an effective methodology for improving and refining the organisational business policies and strategies. The consumers’ end views on trade policy, quality assurance, and service reliability assist the organisation in determining several business parameters, such as pricing and offers, product standards and superiority, consumers’ fulfilment on trading, and payment policy, with a broad aim to achieve total quality of management (TQM) of the business security strategy. We have used the case base analytics of a B2C organisation, where three important parameters affecting the buying behaviour of a consumer are considered. A statistical survey analytic based on consumers’ end evaluation is analysed and weighed. The business security strategy of a B2C organisation depends on the following parameters: service reliability, product quality assurance, trade policies, and buying behaviour. We consider the real-time consumer’s end evaluation data that are to be implemented through the KGAC-framework to build the intelligence for the BI application. The real-time business data sets can be transformed into fuzzified KSs having a unified configuration in the range of [0, 1] to build the intelligence for a BI application.

A computational analysis of a KS for a BI application is presented in Table 3 for further implementation and exploration (S1 File). The KC of a BI application consists of intelligent business parameters (service reliability, product quality assurance, trade policy, and buying behaviour) along with their possible rules of associations, semantic linkages, and dependability standards to cope with the contemporary trends and prospective BI.

**Table 3:** Computational analysis of a BI application in the standard fuzzified range.

Parameter (data type)	Min	Max	Average	Standard deviation	Variance	Standard error mean
Service reliability (real)	0.426	0.708	0.599	0.069	0.0048	0.0029
Product quality assurance (real)	0.152	0.875	0.585	0.239	0.0569	0.0103
Trade policy (real)	0.027	0.931	0.387	0.268	0.072	0.01149
Buying behaviour (real)	0.050	0.925	0.160	0.178	0.0317	0.0077

**Fig 9:**
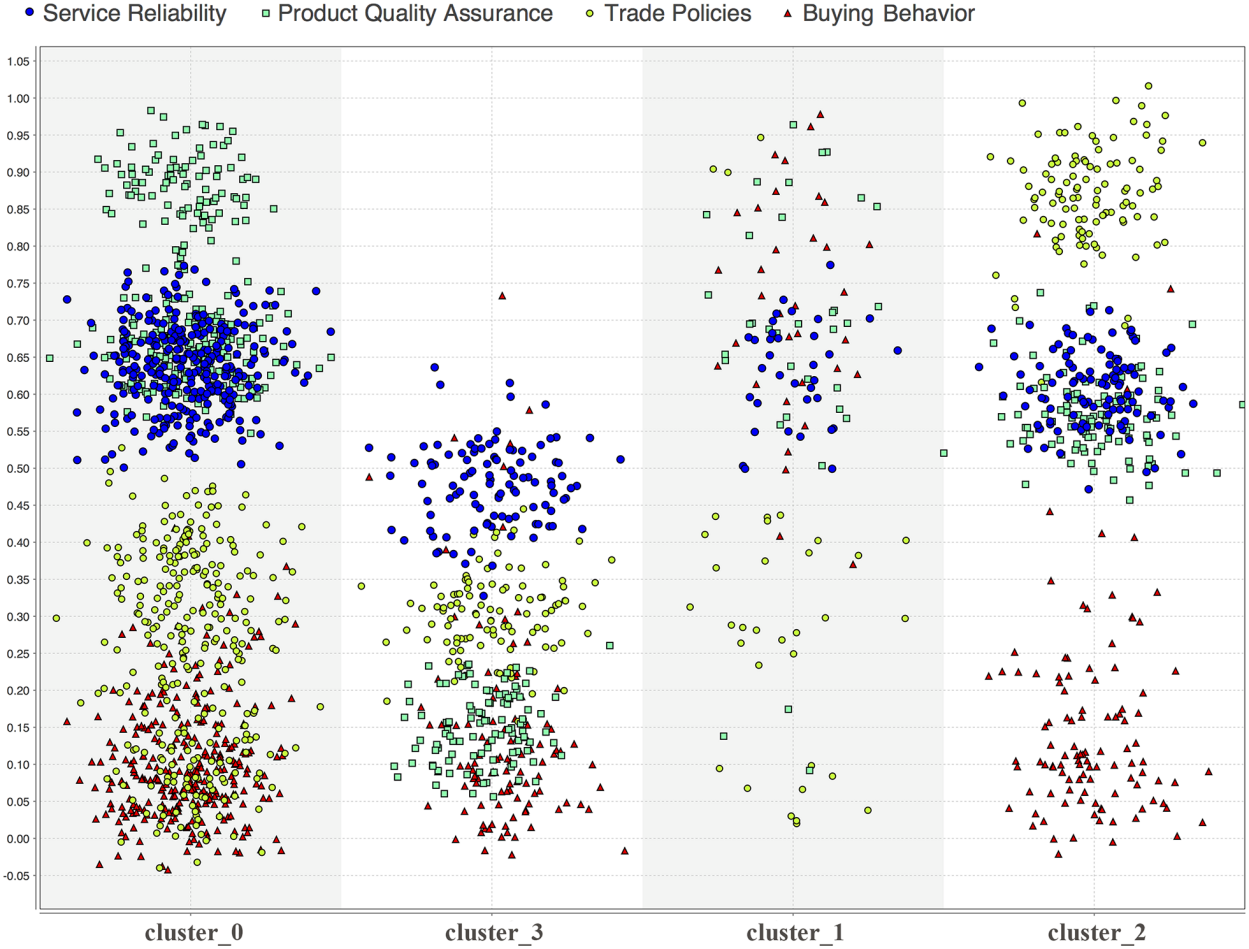
Cluster analysis of knowledge granules associated with the BI parameters of a BI application (cluster_0: poor fit knowledge granules, cluster_3: average fit knowledge granules, cluster_1: good fit knowledge granules, and cluster_2: best fit knowledge granules).

Fig 9 shows the cluster analysis of knowledge granules associated with the intelligence business parameters of a BI application. The graphical analysis indicates several knowledge inferences that are involved with the BI applications. Several other knowledge inferences are also available in fuzzified linguistic terms for the BI application. The clusters are ordered based on the fitness values θ associated with the clusters.

Precision error is a common hazard in the analytic of knowledge granules; it is known as KAP error. A higher KAP error may degrade the appropriateness accuracy for the BI applications. A higher θ value associated with a KS minimises the KAP error for the BI applications. To categorise the quality of KSs in the KCs (KC/θ), we can define three fuzzy membership grades by varying θ values in the range of [0,1] to uniquely distinguish the poor-, average-, good-, and best-fit KSs.

1. $\mu_{kc}(x):x\in X(\theta )\;and\;\theta \;\to \;[0.01,\;0.30];\;iff\;x\;is\;a\;knowledge\;granule\;of\;KC-0\;and\;X\;is\;a\;poor-fit\;KS$
2. $\mu_{kc}(x):x\in X(\theta )\;and\;\theta \;\to \;\left[0.31,\;0.50\right];\;iff\;x\;is\;a\;knowledge\;granule\;of\;KC-3\;and\;X\;is\;an\;average-fit\;KS$
3. $\mu_{kc}(x):x\in X(\theta )\;and\;\theta \;\to \;[0.51,\;0.70];\;iff\;x\;is\;a\;knowledge\;granule\;of\;KC-1\;and\;X\;is\;a\;good-fit\;KS$
4. $\mu_{kc}(x):x\in X(\theta )\;and\;\theta \;\to \;[0.71,\;1.00];\;iff\;x\;is\;a\;knowledge\;granule\;of\;KC-2\;and\;X\;is\;a\;best-fit\;KS$

The neuro-fuzzy structure is capable of extracting the KSs for a multi-rule base system from the BI-statistical business data sets. The neuro-fuzzy environment of MATLAB 2014a also provides the adequate support to implement the neural representations of the GAC-framework using fuzzified data sets to accommodate the fractional values of membership grades to minimise the KAP error [42]. For the analysis, the different θ values associated with three different membership grades may be considered along with the KC to empirically estimate the KAP error. The neuro-fuzzy algorithm can be used as a standard training and testing algorithm for the KGAC framework that calculates the computed outputs to be matched with the desired target output of the training and testing knowledge granules [43–44].

To implement the KGAC framework, we use the least square estimator function evolving with the Gaussian fuzzy membership grade as a learning mechanism, in which the estimator function is intrinsically non-linear in nature. For a non-linear estimator function $f\left(P\right)=\;\alpha \;e^{\beta Q}$, the values of α and are within the fuzzy range {0, 1}, and P and Q are the training knowledge granules. Now, for a training pair (Pi, Qi); i = 1, 2…n, the estimated error $\text{E }\left(\text{Pi},\text{ Qi}\right)\;=\sum_{i=1}^{n}square\left(\text{Pi}-\alpha \;e^{\beta Qi}\right)$, where Pi ϵ f (P). By observing the input/output knowledge granules, predictions are made about behaviours, interactions, and relationships of the framework. Now, based on the above parameters, the KAP error analysis results can be computed as described in Table 4. The θ values of clusters can also be dynamically estimated by quantifying the percentage of outliers present inside that clusters. A greater percentage of outliers present inside a cluster degrades the θ value. For the implementation, the number of nodes in hidden layers is considered to be the number of rules. Table 4 illustrates that the best-fit KC having θ = 0.95 yields the lowest RSME, i.e., 0.01255 and MSE = 0.00016.

**Table 4:** KAP error analysis results.

Number of nodes/rules	θ (Theta values)	MSE (Mean square error)	RSME (Root mean square error)
04	0.01	0.03155	0.17763
04	0.04	0.01475	0.12147
04	0.25	0.00577	0.07597
04	0.40	0.00474	0.06884
04	0.50	0.00408	0.06387
04	0.60	0.00328	0.05724
04	0.80	0.00077	0.02772
04	0.90	0.00029	0.01701
04	0.95	0.00016	0.01255

**Fig 10:**
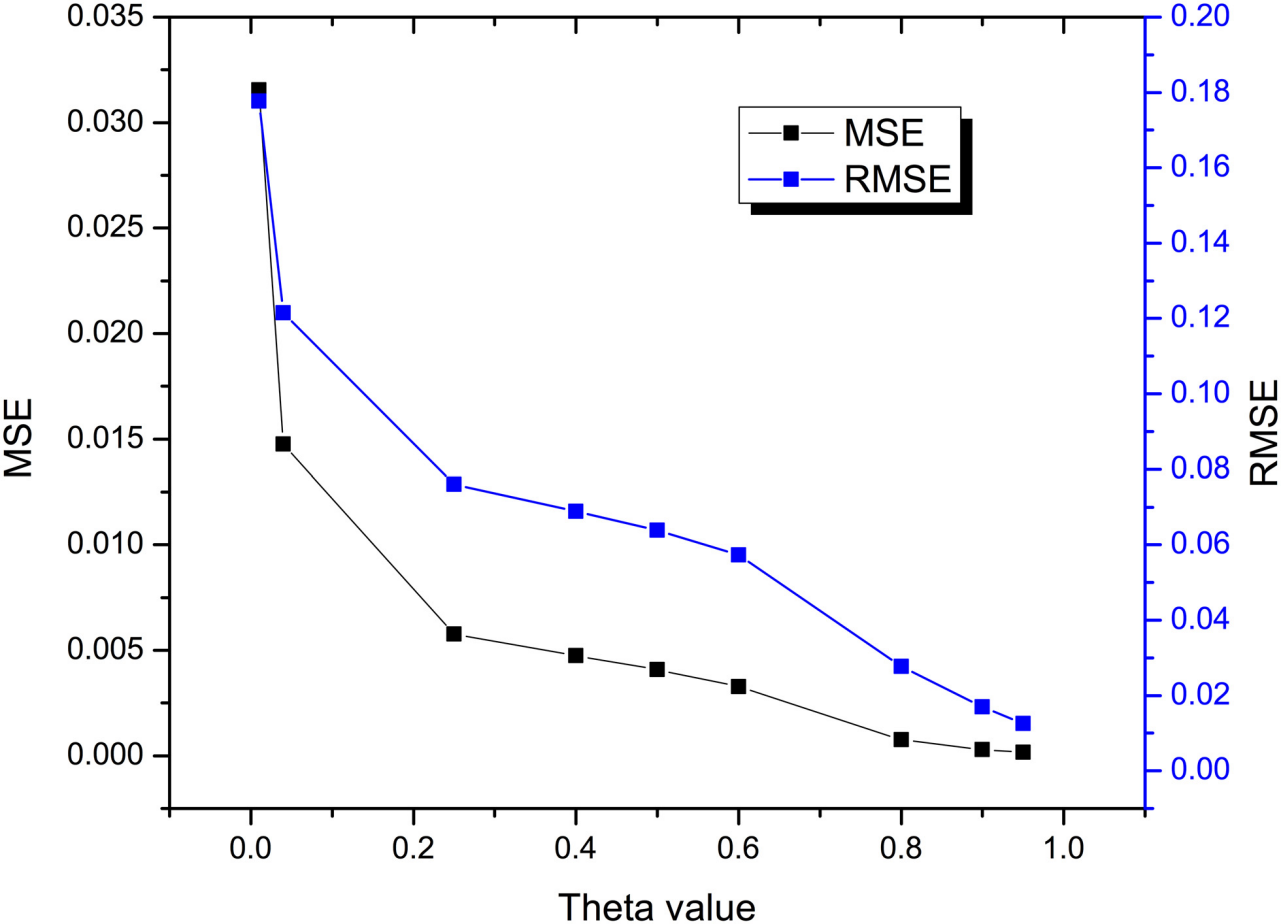
KAP- error analysis result for KGAC framework.

In Fig 10, we present a KAP-error analysis result in terms of estimating the errors (MSE and RSME) for the KGAC-framework. As discussed earlier that KAP-error is inversely propositional to θ, i.e. $\left(KAP-error\;\alpha \;\frac{1}{\theta }\right)$, which indicates that higher the θ values lower is the KAP-error and vice-versa. The graphical illustration identifies more adaptive error deviation between MSE and RSME in between the θ values 0.2 and 0.6. So the effective estimations of errors helps to discover the best fit knowledge cluster that participates in knowledge analytic process so as to generate more precise cognitive decisions and actuations as compared to other poor fit, average fit and good fit knowledge clusters.

Table 5 shows the error comparison result between a standard scaled conjugate gradient (SCG) approach and the KGAC framework approach. MMSE is the minimum mean square error, and MRSME is the minimum root mean square error of the approaches. The computations of both approaches are performed stochastically by analysing the BI application data, whose state is computationally analysed in Table 3. The SCG approach can be effectively used in BI service applications to estimate various business parameters, such as consumers’ buying behaviour, evaluate marketing strategies, devise business security strategies, and assess bank insolvency [45–46]. In our analysis, we obtain MRSME = 0.459184 for the standard SCG approach; this error can be drastically reduced through the KGAC framework approach, which achieves an MRMSE of 0.01255. The analysis demonstrates that the proposed KGAC framework converges more rapidly than the standard SCG approach.

**Table 5:** Error comparison result with standard approach.

Approach	MMSE	MRSME
Standard SCG	0.21085	0.459184
KGAC-framework	0.00016	0.01255

**Fig 11:**
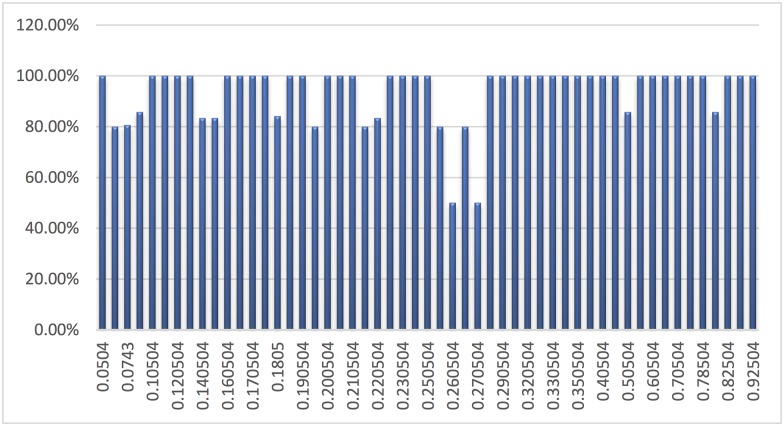
Cluster prediction precision analysis based on the fuzzified input class. The X-axis consists of fuzzified inputs, and the Y-axis consists of the prediction precision percentage.

In Fig 11, we analyse the cluster prediction precision outcomes based on the fuzzified input class. We investigate the uncertainties that may arise during cluster prediction, and numerous probabilistic scenarios are considered to empirically estimate the prediction precisions. We obtain an average cluster prediction precision of 0.9381 in this analysis. We obtain an average cluster prediction error of 0.0619 because several fuzzified input classes could not predict the true cluster class. The degree of uncertainty in the fuzzified input class may affect the predictive outcomes of the proposed KGAC framework. Thus, minimising such errors enables the construction of a robust cluster and sub-cluster organisations of high-dimensional IoT big data for effective knowledge analysis and predictions.

We considered the sensitivity analysis scenario of the potential outcomes of the KGAC framework that can be effectively used for the BI service application. In our analysis, we consider that the BI service application data are higher-dimensional IoT big-data arrays and thereby contribute to the transformation of the analytic of knowledge granules into a business goldmine. Several analyses and explorations that have alliances with the IoT knowledge analytic framework are made. We analyse the semantics and structural analysis of IoT big data that implements cluster and sub-cluster organisation and can be effectively used in business use through the organisation of high-dimensional business data and knowledge based on the θ values associated with the KCs. We perform a KAP-error analysis through a business case analytic for our proposed KGAC framework based on θ values to empirically estimate the MSE and RMSE. We also estimate the error comparison result by comparison with the standard SCG approach.

Thus, based on the needs of the BI applications, the specific KC may be processed for knowledge analytic operations to accomplish various intelligent business tasks, such as planning, forecasting, decision making, and strategy building, for further insights and cognitive actuations. The KGAC framework discovers the KCs having a θ value below the expected threshold to improve the overall effectiveness of the BI application system.

## Conclusions and Further Work

In this work, we propose a KGAC framework for the effective analysis of knowledge granules from IoT big-data arrays for BI applications. An e-KGC mechanism is discussed for the simple clustering of large-scale knowledge granules. The semantic association of knowledge granules inside the clusters and sub-clusters helps represent highly multifaceted decisions that can be used by organisations to develop business intelligence for commercial BI applications. We have presented a detailed discussion of the prospective implementation of a type-2 neuro-fuzzy architecture to achieve the desired level of KAP, industrial applicability, tolerance in precision and uncertainty, and overall functional efficiency. Our analysis and discussion also illustrate the feasibility of discovering knowledge granules with the aim of achieving high KAP. Such a hybrid architecture integrates the good features of neural systems and fuzzy systems with type-2 adaptations to provide higher uncertainty and fault tolerance, better learning ability, better knowledge analytic ability, and better knowledge representation ability compared to a standard fuzzy system by successfully minimising the KAP error. Our framework can help executives and knowledge users generate cognitive decisions, plans, and actuation for the effective monitoring of BI applications.

In the future, we would like to develop a novel re-engineering framework that aims for semantic level knowledge analysis and visualisation-based ontology from IoT big-data arrays for numerous BI applications.

## Supporting Information

S1 File Minimal data set as a text file in fuzzified range [0,1]. (TXT)
